# The interplay between gender identity and sex assigned at birth in adolescents’ health and wellbeing: a cross-sectional study

**DOI:** 10.1186/s12889-025-25954-5

**Published:** 2025-12-24

**Authors:** Gemma Drou-Roget, Marina Bosque-Prous, Judit Rogés, Ester Teixidó-Compañó, Cinta Folch, Esther Sánchez-Ledesma, Gemma Serral, Gloria Perez, Carmen Vives-Cases, Albert Espelt

**Affiliations:** 1 Epi4Health Research Group, Department of Health, Catalan Government, Barcelona, Spain; 2 Department of Community Nursing, Preventive Medicine and Public Health and History of Science, Universitat d’Alacant, Alacant, Spain; 3 Epi4Health Research Group, Department of Psychobiology and Methodology in Health Sciences, Universitat Autònoma de Barcelona (UAB), Barcelona, Bellaterra 08193 Spain; 4 Centro de Investigación Biomédica en Red de Epidemiología y Salud Pública (CIBERESP), Madrid, Spain; 5 Epi4Health Research Group, Department of Epidemiology and Methodology of Social and Health Sciences, Faculty of Health Sciences of Manresa, Universitat de Vic – Universitat Central de Catalunya (UVic-UCC), Manresa, Spain; 6 Centre d’Estudis Epidemiològics sobre les Infeccions de Transmissió Sexual i Sida de Catalunya (CEEISCAT), Institute for Health Science Research Germans Trias i Pujol (IGTP), Can Ruti Campus, Badalona, Spain; 7 Agència de Salut Pública de Barcelona (ASPB), Barcelona, Spain; 8 Institut d’Investigació Biomèdica Sant Pau (IIB SANT PAU), Barcelona, Spain; 9 Faculty of Medicine and Life Sciences, Universitat Pompeu Fabra, Barcelona, Spain

**Keywords:** Sex, Gender, Gender identities, Health inequalities, Adolescents

## Abstract

**Background:**

There is a growing body of evidence on health inequalities according to gender identity, in which sex assigned at birth is often overlooked. The aim of our study was to assess the relevance of examining both gender identity and sex assigned at birth in health population surveys, by comparing the health outcomes, behaviours, and interpersonal relationships in cisgender boys, cisgender girls, transgender and gender expansive (TGE) youth assigned male at birth (AMAB) and TGE youth assigned female at birth (AFAB), in a general population sample of adolescents schooled in Catalonia, Spain.

**Methods:**

Cross-sectional study using a questionnaire in students aged 12–19 years from Catalonia (*N* = 9177) in 2021–2022. We assessed the differences in health outcomes (self-perceived overall health and mental wellbeing), health-related behaviours (quality of diet, physical activity, consumption of tobacco, alcohol and cannabis, and compulsive screen and digital entertainment use), and interpersonal relationships (family relationships, bullying victimization, and sexual violence) in cisgender boys, cisgender girls, TGE AMAB and TGE AFAB, using descriptive statistics and Pearson chi-square tests. We used Poisson regression models with robust variance to examine the associations between the health outcomes and gender identity and sex assigned at birth.

**Results:**

TGE AMAB adolescents had a greater prevalence of worse overall health (PR_adj_=2.01, 95% CI: 1.14–3.52) and mental discomfort (PR_adj_=1.63, 95% CI: 1.20–2.23) than cisgender boys. TGE AFAB adolescents had an increased prevalence of worse overall health (PR_adj_=1.81, 95% CI: 1.42–2.31) than cisgender girls, while the association with mental discomfort was not statistically significant (PR_adj_=1.13, 95% CI: 0.97–1.31). TGE AFAB had an increased prevalence of worse overall health (PR_adj_=1.95, 95% CI: 1.07–3.53) and mental discomfort (PR_adj_=1.47, 95% CI: 1.05–2.05) than TGE AMAB adolescents. Interpersonal relations and health-related behaviours partly explained these associations.

**Conclusions:**

TGE adolescents have worse health outcomes than cisgender peers and, simultaneously, adolescents who were AFAB (cisgender girls and TGE AFAB) have worse health outcomes than those AMAB (cisgender boys and TGE AMAB, respectively). In health research, gender identity should complement, not substitute, sex assigned at birth.

**Supplementary Information:**

The online version contains supplementary material available at 10.1186/s12889-025-25954-5.

## Introduction

There is widespread evidence that there are health inequalities according to different axis of oppression, such as sex, socioeconomic position, ethnicity, age [1], sexual orientation [2], and gender identity [3], among others. Although information systems tend to incorporate questions to monitor those social circumstances, the collection of some of them (like sex or age) is more established than others (like sexual orientation or gender identity) [4].

There is still confusion in the use of “sex” and “gender”, with contributions from different fields of knowledge. In health sciences, sex is generally understood as sex assigned at birth, which can be male, female, or intersex [5]. Gender, from the perspective of feminist sociology, refers to culturally defined roles, responsibilities, attributes, and entitlements associated with being (or being seen as) a woman or man in a given setting, along with the power relations between and among women and men [6]. Since sex assigned at birth entails a certain gender socialization, both terms have been used interchangeably in health sciences, although some researchers suggest to use sex to explain biological differences and gender for social differences [7].

From a gender perspective, girls tend to have worse health outcomes than boys [8]. These inequalities stem from feminine gender norms such as being caring, responsible, physically attractive and submissive, which are related to more internalizing symptoms [9], a complex relationship with nutrition and physical activity [10], and more exposure to abusive experiences [11]; and masculine gender norms such as being stoic, insensitive and violent, which are related to the inability to seek help [12], and to a greater adoption of risky behaviours [6].

Gender is also used in the sense of gender identity, which refers to the components of gender that correspond to a person’s sense of self in relation to their felt and inner sense of gender. The *gender/sex* binary is a belief system that assumes that sex and gender are binary, with two mutually exclusive categories for sex (males/females) and gender (men/women), and that each sex is associated to one gender. This system does not acknowledge that one’s experience of gender might be in congruence (cis) or incongruence (trans) with their sex assigned at birth, or that there are other gender identities beyond man and woman (for instance, non-binary, agender, gender fluid, etc.) [13]. People whose gender identity expands beyond the gender/sex binary are often referred to as *transgender and gender expansive* (TGE) people [3, 14].

In the last two decades, there has been a growing interest in health inequalities regarding gender identity. Compared to cisgender peers, TGE adolescents experience from two to three times increased risk of anxiety, depression, self-harm, suicidal ideation, and suicide attempts [15] and overall poorer health [16]. According to the minority stress model [17], such inequalities are due to the excess of stigma and discrimination that these individuals experience because of their minority and stigmatised social position. These forms of stigma can be individual responses to stigma (such as the adoption of unhealthy behaviours of eating [18], physical activity [19], substance use [20], or problematic internet use [21]); interpersonal (like school bullying [20], family rejection [22], or sexual violence [23]); and structural (at the level of cultural norms and institutional policies that constrain the wellbeing of the stigmatised).

While many studies have documented worse health outcomes, health-related behaviours, and interpersonal relations in TGE than in cisgender adolescents, in many of them the analysis is not stratified by their sex assigned at birth [16, 20–23]. Emerging evidence shows different patterns between TGE adolescents who were assigned male at birth (AMAB) and assigned female at birth (AFAB) [24–27]. Therefore, the aim of this study was to assess the relevance of examining both gender identity and sex assigned at birth in health population surveys, by comparing the health outcomes, behaviours, and interpersonal relationships in cisgender boys, cisgender girls, TGE AMAB and TGE AFAB people, in a general population sample of adolescents schooled in Catalonia, Spain.

## Methods

### Study design and sample

We performed a cross-sectional study using data from the second wave of the DESKcohort project, which monitors data on health and health-related factors in adolescents in Central Catalonia (Spain). All the 98 schools of the region were invited to participate, and 84 of them accepted. Data collection was conducted between October 2021 and May 2022 in the school setting, via a self-administered on-line questionnaire (detailed information available in the protocol paper [28]). The study population consisted in students in 2nd and 4th year of Compulsory Secondary Education (CSE), 2nd year of Post-Compulsory Secondary Education (PCSE), and 2nd year of Intermediate Level Training Cycles (ILTC), aged 12 to 19 years. The final sample consisted of 9177 participants, which represents a 64.4% of the target population [28].

### Variables

The main independent variables were gender identity and sex assigned at birth. Participants were asked which their sex assigned at birth was (male/female) and whether they identified with their sex assigned at birth (yes/no). Those who answered no could indicate their specific gender identity through an open-field question. Participants were grouped in four categories according to both sex assigned at birth and gender identity: cisgender boys (people who were AMAB and identified with this sex), cisgender girls (AFAB and identified with it), TGE AMAB (people who were AMAB but did not identify with this sex) and TGE AFAB (people who were AFAB but did not identify with this sex).

The main dependent variables were self-perceived overall health and mental wellbeing. Self-perceived overall health was assessed by the question “How is your health in general?”, with 5 response options that were dichotomised into good health (excellent, very good or good) and worse health (fair or bad). Mental wellbeing was measured according to the Warwick-Edinburgh Mental Well-Being Scale (WEMWBS) validated for Spanish adolescents [29], which measures positive aspects of mental health in the previous two weeks. It consists of 14 items with a 5-point Likert-type response, and a final score from 14 (mental discomfort) to 70 points (mental wellbeing). In our sample, we obtained a satisfactory global score’s Cronbach’s alpha of 0.896 (CI95%: 0.893–0.900). To assess validity, a Structural equation model with Satorra-Bentler estimation was fitted obtaining good estimations (RSMEA 0.08, CFI 0.90, TLI 0.88 and, SRMR 0.05). For descriptive purposes and in order to provide an estimate of the prevalence of mental discomfort in our population, we dichotomised the scale using the authors’ suggested bench-marking approach, setting a cut-off of ≤ 44 points, which is indicative of possible mild depression according to bench-marking on CES-D (Center for Epidemiologic Studies Depression Scale) [30].

Several health-related behaviours and interpersonal relations variables were also collected using self-reported measures, to further explore differences according to the independent variable and to adjust the final models. Quality of diet was measured according to the Index of a Healthy Alimentation for the Spanish population (IASE) [31]. A score > 80 points is considered a healthy diet and ≤ 80 points indicate an unhealthy diet that needs changes. Physical activity was assessed according to the World Health Organization (WHO) recommendation of doing 1 h per day of moderate to vigorous physical activity. Daily tobacco consumption (yes/no), and cannabis consumption over the last 3 months (yes/no) were collected. Hazardous alcohol consumption was measured according to the validated Alcohol Use Disorders Identification Test (AUDIT-C), using a cut-off of ≥ 3 points for hazardous drinking [32]. Compulsive screen and digital entertainment use were measured with an adaptation of the Compulsive Internet Use Scale (CIUS), with a cut-off of ≥ 28 points for problematic internet use [33].

Regarding interpersonal relations, family relationships were assessed using a single-item question with five response options that were dichotomised (very good or good; regular, bad or very bad). Bullying victimization was based on a question with three items: “In relation to your classmates, have you found yourself in any of these situations in the last year?” (i) You have been treated badly or insulted at school or on the way to school; (ii) you have been hit, attacked and/or threatened at school or on the way to school; and (iii) you have been ostracised or rejected from the group”. For every item, there were five options to answer, from “never” to “4 or more times”. The number of occurrences were added up and people who had experienced any combination of these situations four or more times were considered as having been bullied [34]. Sexual violence was assessed using a multiple response question: “Have you ever suffered any of the following forms of violence?”, with response options “Without physical contact (sexist jokes with sexual content, unwanted continued sexualised staring, exhibitionism, etc.)”, “With physical contact (continuous invasion of own space, unwanted touching, cornering for sexual purposes, etc.)”, “With introduction of objects or body parts in your body, with or without forcing”, “ I haven’t suffered any of those forms of violence”, “I’d rather not answer”. Participants who reported any of the forms of violence were considered to have suffered sexual violence, and participants who preferred not to answer remained an independent analytical category.

Sociodemographic variables were used to describe the sample and adjust the final models. They included age, place of residence (towns of < 30,000 inhabitants, and cities of ≥ 30,000 inhabitants), migration status (from Spain, second-generation migrant if any progenitor was born outside of Spain, first-generation migrant if the adolescent was born outside of Spain, missing if there was no information), self-reported socioeconomic position (low, medium, high) [35], and sexual orientation. For sexual orientation, participants were asked which option they mostly identified with: “Heterosexual (you usually experience other-sex attraction)”; “Homosexual or Lesbian (you usually experience same-sex attraction)”; “Bisexual (you usually experience same-sex and other-sex attraction)”; “I’m questioning/I don’t know”; “I prefer not to answer”; and “Other”. Participants who responded “other” had an additional open response option, and only for descriptive purposes two emerging categories were derived from the most frequent responses: “Pansexual” and “Asexual spectrum”.

### Analysis

We first performed a descriptive analysis of the sample in terms of sociodemographic variables. Second, we explored the differences in the primary outcomes (self-perceived overall health and mental discomfort) and covariates (health-related behaviours and interpersonal relations) according to the independent variable (cisgender boys, cisgender girls, TGE AMAB and TGE AFAB). Differences between groups were assessed using Pearson chi-squared tests for categorical variables.

To analyse the association between the primary outcomes and the independent variable, we estimated the prevalence ratio (PR) with their 95% confidence intervals (CI) using a Poisson regression model with robust variance, as recommended for cross-sectional studies with dichotomous dependent variables [36, 37]. The model was adjusted by age, socioeconomic position, migration status and sexual orientation, as suggested by the Spanish framework to reduce health inequalities [38]. We rotated the reference category to allow pairwise comparisons.

To explore the potential confounding effect of covariates, we then calculated the prevalence of worse overall health and mental discomfort according to the different covariates, with their 95% CI, stratifying by the independent variable. After, we estimated four Poisson regression models with robust variance. In model 1, we estimated the adjusted prevalence ratios (PR) and 95% CI of the primary outcome (worse health or mental discomfort) according to the independent variable, adjusting by age, socioeconomic position, migration status and sexual orientation. In model 2, we added the health-related behaviours and kept only those that were statistically significant. In model 3, we added to model 1 the interpersonal relations variables that were statistically significant. Model 4 included all the variables in model 1, 2 and 3. Lastly, a sensitivity analysis was performed to explore the effect of the 56.7% of the TGE group who did not provide a specific gender identity by excluding these participants, which did not affect the results.

Data analysis was performed with STATA 18.0. Categorical covariates were handled using the i.variable factor-variable notation that generates dummy variables and designates one category as the reference group.

## Results

In our study, 48.4% of the participants were cisgender boys, 49.5% were cisgender girls, 0.7% were transgender and gender expansive (TGE) assigned male at birth (AMAB) and 1.4% were TGE assigned female at birth (AFAB) (Table 1). TGE participants defined themselves in different ways: non-binary, gender fluid, demigender, agender, person, without labels, and questioning or didn’t know (identities composing 34.0% of the TGE group). Only 9.3% of the TGE group identified as trans (9.0% transgender girls and 9.4% transgender boys). The remaining 56.7% of the TGE group did not provide a specific gender identity, percentage that was higher among AMAB (70.1%) than among AFAB (49.6%).

**Table 1:** Sociodemographic description of the sample, stratified by gender identity and sex assigned at birth

	Cisgender boys 4441 (48.4%)	Cisgender girls 4542 (49.5%)		TGE AMAB^a^ 67 (0.7%)	TGE AFAB^a^ 127 (1.4%)
Gender identity; N (%)					
Boy	4441 (100.0%)		-	**-**	12 (9.4%)
Girl	-		4542 (100.0%)	6 (9.0%)	-
Non-binary	-		-	7 (10.4%)	21 (16.5%)
Gender fluid	-		-	2 (3.0%)	16 (12.6%)
Demigender	-		-	0 (0.0%)	3 (2.4%)
Person	-		-	2 (3.0%)	0 (0.0%)
Without labels	-		-	0 (0.0%)	2 (1.6%)
Agender	-		-	0 (0.0%)	2 (1.6%)
I don’t know/questioning	-		-	3 (4.5%)	8 (6.3%)
No identity provided ^b^	-		-	47 (70.1%)	63 (49.6%)
Age, yrs; mean (sd)	15.6 (1.7)		15.6 (1.7)	15.6 (1.8)	15.5 (1.8)
Place of residence; N (%)					
Town (< 30.000 inhabitants)	3134 (70.6%)		3181 (70.0%)	50 (74.6%)	89 (70.1%)
City (≥ 30.000 inhabitants)	1307 (29.4%)		1361 (30.0%)	17 (25.4%)	38 (29.9%)
Migration status; N (%)					
From Spain	3274 (73.7%)		3310 (72.9%)	38 (56.7%)	76 (59.8%)
Second-generation migrant ^c^	715 (16.1%)		780 (17.2%)	16 (23.9%)	27 (21.3%)
First-generation migrant ^c^	293 (6.6%)		275 (6.0%)	5 (7.5%)	10 (7.9%)
Missing	159 (3.6%)		177 (3.9%)	8 (11.9%)	14 (11.0%)
Socioeconomic position; N (%)					
Low	1532 (34.5%)		1581 (34.8%)	22 (32.8%)	57 (44.9%)
Medium	1500 (33.8%)		1468 (32.3%)	20 (29.9%)	30 (23.6%)
High	1409 (31.7%)		1493 (32.9%)	25 (37.3%)	40 (31.5%)
Sexual orientation; N (%)					
Heterosexual	3901 (87.8%)		3094 (68.1%)	39 (58.2%)	34 (26.8%)
Homosexual	97 (2.2%)		90 (2.0%)	3 (4.5%)	9 (7.1%)
Bisexual	153 (3.4%)		697 (15.3%)	11 (16.4%)	38 (29.9%)
Questioning/Don’t know	125 (2.8%)		484 (10.7%)	3 (4.5%)	18 (14.2%)
Prefer not to answer	142 (3.2%)		137 (3.0%)	3 (4.5%)	12 (9.5%)
Pansexual	4 (0.1%)		14 (0.3%)	1 (1.5%)	6 (4.7%)
Asexual spectrum	3 (0.1%)		12 (0.3%)	0 (0.0%)	5 (3.9%)
Other	16 (0.4%)		14 (0.3%)	7 (10.4%)	5 (3.9%)

Alt text: Table displaying the distribution of the sociodemographic in the four groups of study (cisgender boys, cisgender girls, TGE AMAB and TGE AFAB)

^a^TGE AMAB = transgender or gender expansive assigned male at birth; TGE AFAB = transgender or gender expansive assigned female at birth

^b^These participants reported not feeling identified with their sex assigned at birth, but when specifically asked how they identified via an open-field question, they did not provide any answer

^c^Second-generation migrant (any progenitor born outside of Spain) and first-generation migrant (adolescent born outside of Spain)

Participants mean age was around 15 years old, over 70% of participants lived in towns with less than 30,000 inhabitants, the majority were born in Spain (over 56% in every category of the independent variable), and there was representation of different socioeconomic positions (Table 1). Most of the sample identified as heterosexual, although the percentage was higher in cisgender boys (87.8%) than in cisgender girls (68.1%), TGE AMAB (58.2%) and TGE AFAB adolescents (26.8%). The second most common sexual orientation was bisexual, followed by adolescents that were questioning or didn’t know their sexual orientation (Table 1).

**Table 2:** Differences in the primary health outcomes, health-related behaviours, and interpersonal relations, according to gender identity and sex assigned at birth

	Cisgender boys	Cisgender girls	TGE AMAB^a^	TGE AFAB^a^	*p*-value				
	4441 (48.4%)	4542 (49.5%)	67 (0.7%)	127 (1.4%)	Total	Within sex comparison		Between sex comparison	
	*N* (%)	*N* (%)	*N* (%)	*N* (%)		Boys-AMAB	Girls-AFAB	Boys-girls	AMAB- AFAB
**Primary health outcomes**									
Overall health					**< 0.001**	**0.001**	**< 0.001**	**< 0.001**	**0.002**
Good health	4191 (94.4%)	3893 (85.7%)	57 (85.1%)	81 (63.8%)					
Worse health	250 (5.6%)	649 (14.3%)	10 (14.9%)	46 (36.2%)					
Mental wellbeing					**< 0.001**	**< 0.001**	**< 0.001**	**< 0.001**	**0.002**
Mental wellbeing	3662 (82.5%)	2672 (58.8%)	44 (65.7%)	53 (41.7%)					
Mental discomfort	779 (17.5%)	1870 (41.2%)	23 (34.3%)	74 (58.3%)					
**Health-related behaviours**									
Quality of diet (IASE ≤ 80) ^b^					**< 0.001**	0.695	0.101	**< 0.001**	**0.021**
Healthy	248 (5.6%)	504 (11.1%)	3 (4.5%)	20 (15.7%)					
Unhealthy	4193 (94.4%)	4038 (88.9%)	64 (95.5%)	107 (84.3%)					
Physical activity (≥ 1 h/day)					**< 0.001**	0.510	0.522	**< 0.001**	**0.010**
Yes	2564 (57.7%)	1700 (37.4%)	36 (53.7%)	44 (34.6%)					
No	1877 (42.3%)	2842 (62.6%)	31 (46.3%)	83 (65.4%)					
Daily tobacco consumption					**< 0.001**	0.797	0.727	**< 0.001**	0.284
No	4211 (94.8%)	4187 (92.2%)	64 (95.5%)	116 (91.3%)					
Yes	230 (5.2%)	355 (7.8%)	3 (4.5%)	11 (8.7%)					
Cannabis consumption (last 30 days)					0.366	0.298	0.239	0.369	0.795
No	4178 (94.1%)	4293 (94.5%)	61 (91.0%)	117 (92.1%)					
Yes	263 (5.9%)	249 (5.5%)	6 (9.0%)	10 (7.9%)					
Risk alcohol consumption (AUDIT-C ≥ 3) ^c^					**0.035**	0.544	0.139	**0.011**	0.957
No	3302 (74.4%)	3269 (72.0%)	52 (77.6%)	99 (78.0%)					
Yes	1139 (25.6%)	1273 (28.0%)	15 (22.4%)	28 (22.0%)					
Compulsive screen and digital entertainment use (CIUS ≥ 28) ^d^					**< 0.001**	0.099	0.322	**< 0.001**	0.317
No	3659 (82.4%)	3258 (71.7%)	50 (74.6%)	86 (67.7%)					
Yes	782 (17.6%)	1284 (28.3%)	17 (25.4%)	41 (32.3%)					
**Interpersonal relationships**									
Family relationships					**< 0.001**	**< 0.001**	**< 0.001**	**< 0.001**	**0.040**
Very good/good	3956 (89.1%)	3507 (77.2%)	50 (74.6%)	76 (59.8%)					
Regular/bad/very bad	485 (10.9%)	1,035 (22.8%)	17 (25.4%)	51 (40.2%)					
Bullying victimization					**< 0.001**	**< 0.001**	**< 0.001**	**< 0.001**	0.571
No	3809 (85.8%)	3686 (81.2%)	47 (70.1%)	84 (66.1%)					
Yes	632 (14.2%)	856 (18.8%)	20 (29.9%)	43 (33.9%)					
Any sexual violence					**< 0.001**	**< 0.001**	0.174	**< 0.001**	**< 0.001**
No	3885 (87.5%)	1976 (43.5%)	45 (67.1%)	45 (35.4%)					
Yes	399 (9.0%)	2,380 (52.4%)	16 (23.9%)	75 (59.1%)					
Prefer not to answer	157 (3.5%)	186 (4.1%)	6 (9.0%)	7 (5.5%)					

Differences between groups were assessed using Pearson chi-squared tests; statistically significant differences are highlighted in **bold**

^a^TGE AMAB = transgender or gender expansive assigned male at birth; TGE AFAB = transgender or gender expansive assigned female at birth

^b^Spanish Healthy Alimentation Index (IASE), using a cut-off of ≤ 80 points for unhealthy diet

^c^Alcohol Use Disorders Identification Test (AUDIT-C), using a cut-off of ≥ 3 points for hazardous drinking

^d^Compulsive Internet Use Scale (CIUS), with a cut-off of ≥ 28 points for problematic internet use

Alt text: Table displaying the differences of the primary outcomes and covariates according to the four categories of the independent variable (cisgender boys, cisgender girls, TGE AMAB and TGE AFAB), with p-values for the comparisons within sex (cisgender boys vs. TGE AMAB, and cisgender girls vs. TGE AFAB) and between sex (cisgender boys vs. cisgender girls, and TGE AMAB vs. TGE AFAB)

The primary health outcomes, as well as health-related behaviours and interpersonal relationships covariates showed differences according to gender identity and sex assigned at birth (Table 2). Worse overall health was more frequent among TGE AFAB (36.2%), followed by TGE AMAB and cisgender girls (14.9% and 14.3%) and finally cisgender boys (5.6%), with statistically significant differences overall, within sex (comparing cisgender boys vs. TGE AMAB, and cisgender girls vs. TGE AFAB) and between sex (comparing cisgender boys vs. girls, and TGE AMAB vs. AFAB). The pattern was similar for mental discomfort, which was more frequent among TGE AFAB (58.3%), followed by cisgender girls (41.2%), TGE AMAB (34.3%) and cisgender boys (17.5%), with statistically significant differences overall, within sex and between sex.

All health-related behaviours, except cannabis consumption in the last 30 days, showed statistically significant differences between groups (Table 2). There was more daily tobacco consumption in TGE AFAB (8.7%) and cisgender girls (7.8%) than in cisgender boys (5.2%) and TGE AMAB (4.5%); more risk alcohol consumption in cisgender girls (28.0%) than in cisgender boys (25.6%), TGE AMAB (22.4%) and TGE AFAB (22.0%), and more compulsive screen and digital entertainment use in TGE AFAB (32.3%) and in cisgender girls (28.3%) than in TGE AMAB (25.4%) and cisgender boys (17.6%). However, these differences were only statistically significant between cisgender boys and cisgender girls. For quality of diet and physical activity, statistically significant differences were also found between TGE AMAB and TGE AFAB: more TGE AMAB had an unhealthy diet than TGE AFAB (95.5% vs. 84.3%, p-value = 0.021), and more TGE AFAB did insufficient physical activity than TGE AMAB (65.4% vs. 46.3%, p-value < 0.001).

All variables on interpersonal relations showed worse results in TGE than in cisgender participants. Bullying victimization was more frequently reported by TGE AFAB (33.9%), TGE AMAB (29.9%), cisgender girls (18.8%) and cisgender boys (14.2%), with statistically significant differences between cisgender and TGE participants (p-value < 0.001) but similar results between TGE AMAB and AFAB. A similar pattern was observed for regular/bad/very bad family relationships, although in this case differences were also found between TGE AMAB and AFAB (25.4% vs. 40.2%, p-value = 0.040). Sexual violence was more frequent amongst TGE AFAB (59.1%) and cisgender girls (52.4%) than in TGE AMAB (23.9%) and in cisgender boys (9.0%), with statistically significant differences between cisgender boys and girls (p-value < 0.001), between TGE AMAB and TGE AFAB (p-value < 0.001), and between cisgender boys and TGE AMAB (p-value < 0.001), but not between cisgender girls and TGE AFAB.

Table 3 shows the association between the primary outcomes and the independent variable, adjusted by age, socioeconomic position, migration status, and sexual orientation. In comparison with cisgender boys, all other groups had an increased prevalence of worse overall health and mental discomfort. TGE AMAB had a greater prevalence of worse overall health (PR_adj_=2.01, 95% CI: 1.14–3.52) and mental discomfort (PR_adj_=1.63, 95% CI: 1.20–2.23) than cisgender boys. TGE AFAB had an increased prevalence of worse overall health (PR_adj_=1.81, 95% CI: 1.42–2.31) than cisgender girls, while the association with mental discomfort was not statistically significant (PR_adj_=1.13, 95% CI: 0.97–1.31). When comparing TGE groups, TGE AFAB had an increased prevalence of worse overall health (PR_adj_=1.95, 95% CI: 1.07–3.53) and mental discomfort (PR_adj_=1.47, 95% CI: 1.05–2.05) than TGE AMAB. Finally, there were differences between cisgender girls compared to cisgender boys in worse overall health (PR_adj_=2.16, 95% CI: 1.87–2.49) and mental discomfort (PR_adj_=2.13, 95% CI: 1.98–2.29), but not compared to TGE AMAB (PR_adj_=1.07, 95% CI: 0.62–1.87; and PR_adj_=1.30, 95% CI: 0.96–1.77, respectively).

**Table 3:** Analysis of the association of worse overall health (A) and mental discomfort (B) with gender identity and sex assigned at birth

A. Worse overall health									
		**Ref: cisgender boys**		**Ref: cisgender girls**		**Ref: TGE AMAB**		**Ref: TGE AFAB**	
	***N***	**PR**_**adj**_^**a**^	**95% CI**	**PR**_**adj**_^**a**^	**95% CI**	**PR**_**adj**_^**a**^	**95% CI**	**PR**_**adj**_^**a**^	**95% CI**
Gender identity									
Cisgender boys	4441	1.00		**0.46**	**(0.40 - 0.54)**	**0.50**	**(0.28 - 0.88)**	**0.26**	**(0.20 - 0.34)**
Cisgender girls	4542	**2.16**	**(1.87 - 2.49)**	1.00		1.07	(0.62 - 1.87)	**0.55**	**(0.43 - 0.71)**
TGE AMAB ^b^	67	**2.01**	**(1.14 - 3.52)**	0.93	(0.54 - 1.62)	1.00		**0.51**	**(0.28 - 0.93)**
TGE AFAB ^b^	127	**3.90**	**(2.97 - 5.12)**	**1.81**	**(1.42 - 2.31)**	**1.95**	**(1.07 - 3.53)**	1.00	

B. Mental discomfort									
		**Ref: cisgender boys**		**Ref: cisgender girls**		**Ref: TGE AMAB**		**Ref: TGE AFAB**	
	**N**	**PR**_**adj**_^**a**^	**95% CI**	**PR**_**adj**_^**a**^	**95% CI**	**PR**_**adj**_^**a**^	**95% CI**	**PR**_**adj**_^**a**^	**95% CI**
Gender identity									
Cisgender boys	4441	1.00		**0.47**	**(0.44 - 0.51)**	**0.61**	**(0.45 - 0.84)**	**0.42**	**(0.36 - 0.49)**
Cisgender girls	4542	**2.13**	**(1.98 - 2.29)**	1.00		1.30	(0.96 - 1.77)	0.89	(0.77 - 1.03)
TGE AMAB ^b^	67	**1.63**	**(1.20 - 2.23)**	0.77	(0.57 - 1.04)	1.00		**0.68**	**(0.49 - 0.95)**
TGE AFAB ^b^	127	**2.40**	**(2.04 - 2.82)**	1.13	(0.97 - 1.31)	**1.47**	**(1.05 - 2.05)**	1.00	

Every column provides an estimated prevalence ratio rotating the reference category. Prevalence ratios that are statistically significant compared to the reference category are highlighted in **bold**

^a^Models A and B were adjusted by age, socioeconomic position, migration status, and sexual orientation

^b^TGE AMAB = transgender or gender expansive assigned male at birth; TGE AFAB = transgender or gender expansive assigned female at birth

Alt text: Two sub-tables labelled 3 A and 3B. They display the adjusted prevalence ratios and 95% confidence intervals of worse overall health (3 A) and mental discomfort (3B) in cisgender boys, cisgender girls, TGE AMAB, and TGE AFAB, with each column rotating the reference category

The primary outcomes were also associated with the health-related behaviours and interpersonal relations covariates (Supplementary Tables 1 and 2). The effect of these covariates in the association between the primary outcomes and the independent variable was explored in Table 4. The association between being TGE AFAB and worse overall health compared to cisgender boys (PR_adj_=3.90, 95% CI: 2.97–5.12) decreased considerably when adding relations (PR_adj_=2.66, 95% CI: 2.04–3.48) and, to a lesser extent, when adding behaviours (PR_adj_=3.44, 95% CI: 2.60–4.55) (Table 4 A, models 1–3). Similarly, the association between being TGE AFAB and mental discomfort (PR_adj_=2.40, 95% CI: 2.04–2.82) decreased with relations (PR_adj_=1.78, 95% CI: 1.5–2.10) to a greater extent than with behaviours (PR_adj_=2.20, 95% CI: 1.87–2.59) (Table 4B, models 1–3). The same pattern was observed for the association between cisgender girls and TGE AMAB and both health outcomes. In TGE AMAB, the association with worse overall health was no longer significant after inclusion of behaviours and relations (Table 4 A, models 2 and 3) and mental discomfort lost significance after inclusion of relations (Table 4 B, model 3). Notably, having poor family relations was the covariate more strongly associated with worse overall health (PR_adj_=2.37, 95% CI: 2.09–2.69) and mental discomfort (PR_adj_=1.95, 95% CI: 1.83–2.07) (Supplementary Tables 3 and 4). Overall, these results suggest an important effect of behaviours and relations, especially the latter, in explaining the differences in overall health and mental discomfort according to sex assigned at birth and gender identity.

**Table 4:** Analysis of the association of worse overall health (A) or mental discomfort (B) with gender identity and sex assigned at birth, and the effect of health-related behaviours and interpersonal relationships

A. Worse overall health									
		**Model 1** ^a^		**Model 2 (behaviours)** ^b^		**Model 3 (relations)** ^d^		**Model 4** ^e^	
	**N**	**PR**_**adj**_	**95% CI**	**PR**_**adj**_	**95% CI**	**PR**_**adj**_	**95% CI**	**PR**_**adj**_	**95% CI**
Gender identity									
Cisgender boys	4441	1.00		1.00		1.00		1.00	
Cisgender girls	4542	**2.16**	**(1.87 - 2.49)**	**1.89**	**(1.63 - 2.19)**	**1.73**	**(1.48 - 2.02)**	**1.61**	**(1.37 - 1.89)**
TGE AMAB ^f^	67	**2.01**	**(1.14 - 3.52)**	1.74	(0.93 - 3.25)	1.53	(0.85 - 2.75)	1.41	(0.74 - 2.71)
TGE AFAB ^f^	127	**3.90**	**(2.97 - 5.12)**	**3.44**	**(2.60 - 4.55)**	**2.66**	**(2.04 - 3.48)**	**2.62**	**(2.00 - 3.42)**

B. Mental discomfort									
		**Model 1** ^a^		**Model 2 (behaviours)** ^c^		**Model 3 (relations)** ^d^		**Model 4** ^e^	
	**N**	**PR**_**adj**_	**95% CI**	**PR**_**adj**_	**95% CI**	**PR**_**adj**_	**95% CI**	**PR**_**adj**_	**95% CI**
Gender identity									
Cisgender boys	4441	1.00		1.00		1.00		1.00	
Cisgender girls	4542	**2.13**	**(1.98 - 2.29)**	**1.97**	**(1.82 - 2.12)**	**1.79**	**(1.66 - 1.94)**	**1.71**	**(1.58 - 1.85)**
TGE AMAB ^f^	67	**1.63**	**(1.20 - 2.23)**	**1.57**	**(1.14 - 2.17)**	1.34	(0.98 - 1.85)	1.34	(0.97 - 1.85)
TGE AFAB ^f^	127	**2.40**	**(2.04 - 2.82)**	**2.20**	**(1.87 - 2.59)**	**1.78**	**(1.51 - 2.10)**	**1.73**	**(1.47 - 2.04)**

Prevalence ratios (PR) that are statistically significant are highlighted in **bold**

^a^ Models 1 A and B were adjusted by age, socioeconomic position, migration status, and sexual orientation

^b^Model 2 A was adjusted by the variables in model 1 and quality of diet, physical activity, daily tobacco consumption, last month cannabis consumption, and compulsive screen and digital entertainment use

^c^Model 2B was adjusted by the variables in model 1 and quality of diet, physical activity, last month cannabis consumption, and compulsive screen and digital entertainment use

^d^Models 3 A and 3B were adjusted by the variables in model 1 and family relationships, bullying victimization and any sexual violence

^e^Models 4 A and 4B were adjusted by all the variables in the previous models

^f^TGE AMAB = transgender or gender expansive assigned male at birth; TGE AFAB = transgender or gender expansive assigned female at birth

Alt text: Two sub-tables labelled 4 A and 4B. Table 4 A displays 4 models (models A1 to A4) that estimate the adjusted prevalence ratios and 95% confidence intervals of worse overall health in cisgender girls, TGE AMAB, and TGE AFAB, compared to cisgender boys; each of the models incorporating new adjustment variables to the previous one. Table 4B follows the same structure as Table 4 A, but here the models present presenting the adjusted prevalence ratios and 95% confidence intervals of mental discomfort in cisgender girls, TGE AMAB, and TGE AFAB, compared to cisgender boys

## Discussion

This study identified differences in self-perceived overall health and mental discomfort among cisgender boys, cisgender girls, TGE AMAB and TGE AFAB adolescents, and that interpersonal relationships and, to a lesser extent, health-related behaviours, partly explained the differences between groups.

### Worse health outcomes in TGE adolescents: the minority stress model

Our results show that TGE adolescents (both AMAB and AFAB) display worse self-perceived overall health and mental discomfort than their cisgender peers (boys and girls respectively) [16, 26]. Notably, these results were not confounded by a major proportion of AFAB in the TGE group, as in other studies that do not consider sex assigned at birth [16]. Differences according to gender identity have been interpreted through the minority stress model, which posits that mental health inequalities are due to the excess of stigma and discrimination that TGE individuals experience because of their minority and stigmatised social position [17]. In line with this model, in our study TGE participants reported worse family relations, more bullying victimization and more sexual violence than cisgender participants [20, 22, 23], both AMAB and AFAB. Moreover, these negative interpersonal experiences partly explained the associations between TGE AFAB and TGE AMAB and mental discomfort.

The associations between TGE and mental discomfort were also partly explained, although to a lesser extent, by health-related behaviours. These findings too fit in the minority stress model, as unhealthy or risk behaviours can be adopted to cope with the experienced discrimination and stigma [17, 39]. Although the minority stress model was developed to explain mental health inequalities, the uptake of unhealthy coping strategies [18–21], together with the increased mental discomfort, which is a health outcome in itself, could also explain the worse self-perceived overall health in TGE adolescents [16, 40].

It should be noted that in our study most of the TGE sample identified in the non-binary spectrum. Therefore, our results might not be generalizable to the transgender population. Other studies report worse health outcomes among non-binary adolescents than transgender adolescents [26]. It has been suggested that non-binary adolescents face additional stressors like invalidation of their identity and pressure to choose one identity within the binary system (boy or girl), sometimes even from the LGBTQIA + community [41]. This resembles the invalidation experience of bisexual adolescents, people who are also forced to choose within a binary model of desire [42], leading to worse health outcomes in bisexual than heterosexual or gay/lesbian adolescents [43]. Overall, these studies suggest that there might be more societal resistance toward identities that challenge the sex-gender-sexuality binarism deeply entrenched in our society [44], and that more targeted studies for specific TGE subgroups can provide interesting nuances.

### Similarities across gender identities due to sex assigned at birth: binary gender socialization

Our results showed worse health outcomes in TGE AFAB than in TGE AMAB, in line with other studies [25–27]. This highlights the importance of collecting sex assigned at birth even when people do not identify with it. Notably, TGE groups showed some similarities to their cisgender peers with the same sex assigned at birth. For instance, TGE AFAB and cisgender girls had similar levels of mental discomfort, which were higher than in adolescents who were assigned male at birth (TGE AMAB and cisgender boys). Moreover, sexual violence, which is an outcome with a very clear gender component, was greatly experienced by cisgender girls and TGE AFAB, at similar levels between them, which relates to the fact that people who are seen by society as women are treated according to a system that legitimates violence against women [6, 11].

Additionally, behaviours like quality of diet and physical activity presented differences between TGE AMAB and TGE AFAB, like those observed between cisgender boys and girls. Part of the difference in alimentation and physical activity between boys and girls can be explained by different normative expectations of the body, with girls generally aiming to have a slender and smaller body and boys concerned with muscularity [6]. Moreover, there are different barriers and facilitators to physical activity for adolescent boys and girls [45].

We hypothesize that these results respond to the fact that social norms and contextual factors shape adolescents to fit into certain gender norms determined by sex assigned at birth [46, 47]. Even though TGE adolescents do not identity with their sex assigned at birth, they are still socialised accordingly from early childhood, which would explain the similarities in some behaviours and outcomes between cisgender girls and TGE AFAB, and between cisgender boys and TGE AMAB. Again, it should be noted that most of our TGE sample was non-binary; in the case of transgender adolescents, they might also have the pressure to conform to their felt gender identity in what is known as “passing” [48]. Nevertheless, our results suggest that sex assigned at birth has implications beyond the biological aspect [7], as it is the basis for a binary socialization process that has many other impacts on health [6].

### Limitations and strengths

One of the limitations of our study was the collection of some multidimensional variables, such as gender identity [13, 49] or sexual orientation [50], with simple questions. General population surveys do not aim to study those constructs in depth and therefore do not incorporate a wider battery of questions to fully capture their complexity; however, we argue that routinely including simpler questions that can be used as a proxy for those constructs is still relevant to account for existing health inequalities in general population [51]. A strength of the study is that it shows how these simplified constructs are sensitive enough to highlight health inequalities in a general population sample.

Another limitation regarding gender identity was the grouping of different gender identities under the umbrella term *transgender and gender expansive* adolescents, partly motivated by a small sample size. The more frequent gender identities were within the non-binary spectrum, with only a small fraction of the group identifying as transgender boys and girls. Therefore, although our results show a tendency for the broad TGE group, they might obscure the specificities of certain subgroups like transgender boys and girls. A striking 56.7% of the TGE group did not inform a specific gender identity beyond reporting they did not identify with their sex assigned at birth. However, the sensitivity analysis yielded the same results when excluding these participants. Although insightful information is sure to be obtained from ad hoc research with sampling strategies that over-recruit TGE youth to analyse within group differences, we believe it is valuable that general population studies account for diversity and report inequalities derived from gender identity and sex assigned at birth.

Finally, some additional considerations should be noted. First, with regard to the sample, even though it is not strictly population-based, we refer to prevalences throughout the manuscript since 85.7% of the schools participated (without any selection bias related to territory or socioeconomic position), with a participation of 64.6% of the target population [28]. Second, since the variables were self-reported, we cannot rule a certain social-desirability bias in the response of the survey, although the fact that it was self-administered digitally and anonymously might reduce this bias. Notably, most covariates were dichotomised potentially losing nuances but prioritizing stability in modelling the main outcomes. Third, since we performed multiple comparisons of the primary outcomes (worse health and mental discomfort) and covariates (health-related behaviours and interpersonal relations) according to the independent variable, this could have led to type I errors. We did not apply a formal multiple-comparison correction because we considered to have only two primary outcomes, and the covariates were well justified in line with the Social Determinants of Health Model (which includes lifestyle factors and social and community networks) and empirical evidence showing differences in these variables [18–23]. Yet, we acknowledge that the comparison between groups in the bivariate analysis should be interpreted with some caution, while the final models do not have this risk since covariates are used for adjustment, not as outcomes. Finally, the cross-sectional design does not allow to infer causality, nor mediating effects of health-related behaviours and interpersonal relations; still, it does show that these variables act as confounders of the observed associations, with longitudinal studies supporting this interpretation [52].

## Conclusion

Although transgender and gender expansive (TGE) adolescents are a small fraction of the general population, health research should be sensitive to gender identities that do not conform with the gender/sex binary, since they have the right to express their gender identity, and they also might face an increased burden of discrimination and health problems. Nevertheless, gender identity should complement but not substitute sex assigned at birth, as both variables have intersecting impacts on health, and female sex assigned at birth is associated with worse health outcomes among cisgender but also TGE adolescents.

## Supplementary Information

Supplementary Material 1

## Data Availability

Both the DESKcohort project and its associated databases are the intellectual property of all members of the Epi4health consortium. This consortium includes the Universitat Autònoma de Barcelona, the Universitat de Vic–Universitat Central de Catalunya, the Universitat Oberta de Catalunya, the Centre d’Estudis Epidemiològics sobre el VIH/SIDA de Catalunya, the Agència de Salut Pública de Barcelona, and the Agència de Salut Pública de Catalunya. The original databases are safeguarded by the Fundació Universitària del Bages. Access to the databases can be requested by contacting the corresponding author of the study. https://deskcohort.cat/en/2nd-wave-2021-2022-schoolyear/.

## Abbreviations

TGE: Transgender and gender expansive
AFAB: Assigned female at birth
AMAB: Assigned male at birth
