# Supplementary material for: The interplay between gender identity and sex assigned at birth in adolescents’ health and wellbeing: a cross-sectional study
Source: BMC Public Health. 2025 Dec 24;26:344. doi: 10.1186/s12889-025-25954-5 (PMC12849083; doi:10.1186/s12889-025-25954-5)
Supplement: Supplementary file 1 — Supplementary Material 1 [file 12889_2025_25954_MOESM1_ESM.docx]

**Supplementary Table 1. Prevalence of worse overall health (% and 95% CI) according to health-related behaviours and interpersonal relationships covariates, stratified by gender identity and sex assigned at birth.**

|  | **Cisgender boys** | | **Cisgender girls** | | **TGE AMAB ^a^** | | **TGE AFAB ^a^** | |  |  |  |  |  |  |  |  |
| --- | --- | --- | --- | --- | --- | --- | --- | --- | --- | --- | --- | --- | --- | --- | --- | --- |
|  | **N** | **% (95% CI)** | **N** | **% (95% CI)** | **N** | **% (95% CI)** | **N** | **% (95% CI)** |  |  |  |  |  |  |  |  |
| **General** | 4441 | 5.6 (5-6.3) | 4542 | 14.3 (13.3-15.3) | 67 | 14.9 (8.2-25.6) | 127 | 36.2 (28.3-44.9) |  |  |  |  |  |  |  |  |
| **Health behaviours** | | | | | | | | |  |  |  |  |  |  |  |  |
| **Quality of diet (IASE≤80)** ^b^ |  |  |  |  |  |  |  |  |  |  |  |  |  |  |  |  |
| Healthy | 248 | 2.4 (1.1-5.3) | 504 | **10.5 (8.1-13.5)** | 3 | 33.3 (4.2-85.2) | 20 | 25.0 (10.7-48.1) |  |  |  |  |  |  |  |  |
| Unhealthy | 4193 | 5.8 (5.1-6.6) | 4038 | **14.8 (13.7-15.9)** | 64 | 14.1 (7.4-25.1) | 107 | 38.3 (29.5-47.9) |  |  |  |  |  |  |  |  |
| **Physical activity (≥1h/day)** |  |  |  |  |  |  |  |  |  |  |  |  |  |  |  |  |
| Yes | 2564 | **3.5 (2.9-4.3)** | 1700 | **10.6 (9.2-12.1)** | 36 | 13.9 (5.8-29.7) | 44 | 25.0 (14.3-39.9) |  |  |  |  |  |  |  |  |
| No | 1877 | **8.5 (7.3-9.9)** | 2842 | **16.5 (15.2-17.9)** | 31 | 16.1 (6.8-33.8) | 83 | 42.2 (32.0-53.1) |  |  |  |  |  |  |  |  |
| **Daily tobacco consumption** |  |  |  |  |  |  |  |  |  |  |  |  |  |  |  |  |
| No | 4211 | **5.2 (4.5-5.9)** | 4187 | **13.0 (12.0-14.1)** | 64 | 14.1 (7.4-25.1) | 116 | 33.6 (25.6-42.8) |  |  |  |  |  |  |  |  |
| Yes | 230 | **14.3 (10.4-19.5)** | 355 | **29.3 (24.8-34.2)** | 3 | 33.3 (4.2-85.2) | 11 | 63.6 (33.6-85.8) |  |  |  |  |  |  |  |  |
| **Cannabis consumption (last 30 days)** |  |  |  |  |  |  |  |  |  |  |  |  |  |  |  |  |
| No | 4178 | **5.3 (4.7-6.0)** | 4293 | **13.3 (12.3-14.3)** | 61 | 14.8 (7.8-26.2) | 117 | 33.3 (25.3-42.4) |  |  |  |  |  |  |  |  |
| Yes | 263 | **11.0 (7.8-15.4)** | 249 | **31.7 (26.2-37.8)** | 6 | 16.7 (2.2-64.1) | 10 | 70.0 (37.3-90.1) |  |  |  |  |  |  |  |  |
| **Risk alcohol consumption (AUDIT-C≥3) ^c^** |  |  |  |  |  |  |  |  |  |  |  |  |  |  |  |  |
| No | 3302 | 5.3 (4.6-6.2) | 3269 | **12.5 (11.4-13.7)** | 52 | 13.5 (6.5-25.9) | 99 | 33.3 (24.7-43.3) |  |  |  |  |  |  |  |  |
| Yes | 1139 | 6.5 (5.2-8.1) | 1273 | **18.9 (16.8-21.1)** | 15 | 20.0 (6.4-47.6) | 28 | 46.4 (29.0-64.7) |  |  |  |  |  |  |  |  |
| **Compulsive screen and digital entertainment use (CIUS≥28) ^d^** |  |  |  |  |  |  |  |  |  |  |  |  |  |  |  |  |
| No | 3659 | **4.7 (4.1-5.4)** | 3258 | **10.8 (9.8-11.9)** | 50 | 14.0 (6.7-26.9) | 86 | 37.2 (27.6-48.0) |  |  |  |  |  |  |  |  |
| Yes | 782 | **10.0 (8.1-12.3)** | 1284 | **23.1 (20.9-25.5)** | 17 | 17.6 (5.7-43.3) | 41 | 34.1 (21.3-49.9) |  |  |  |  |  |  |  |  |
| **Interpersonal relations** | | | | | | | | |  |  |  |  |  |  |  |  |
| **Family relationships** |  |  |  |  |  |  |  |  |  |  |  |  |  |  |  |  |
| Good or very good | 3956 | **4.5 (3.9-5.2)** | 3507 | **9.6 (8.6-10.6)** | 50 | 10.0 (4.2-22.2) | 76 | **19.7 (12.2-30.3)** |  |  |  |  |  |  |  |  |
| Regular/bad/very bad | 485 | **14.8 (12.0-18.3)** | 1035 | **30.2 (27.5-33.1)** | 17 | 29.4 (12.6-54.7) | 51 | **60.8 (46.8-73.2)** |  |  |  |  |  |  |  |  |
| **Bullying victimization** |  |  |  |  |  |  |  |  |  |  |  |  |  |  |  |  |
| No | 3809 | **4.6 (4.0-5.3)** | 3686 | **12.3 (11.3-13.4)** | 47 | 10.6 (4.4-23.4) | 84 | 29.8 (20.9-40.5) |  |  |  |  |  |  |  |  |
| Yes | 632 | **11.7 (9.4-14.5)** | 856 | **22.8 (20.1-25.7)** | 20 | 25.0 (10.6-48.3) | 43 | 48.8 (34.3-63.6) |  |  |  |  |  |  |  |  |
| **Any sexual violence** |  |  |  |  |  |  |  |  |  |  |  |  |  |  |  |  |
| No | 3885 | **4.9 (4.2-5.6)** | 1976 | **10.3 (9.0-11.7)** | 45 | 13.3 (6.0-27.0) | 45 | 24.4 (14.0-39.1) |  |  |  |  |  |  |  |  |
| Yes | 399 | **11.8 (9.0-15.3)** | 2380 | **17.2 (15.7-18.8)** | 16 | 18.8 (6.0-45.3) | 75 | 42.7 (31.9-54.2) |  |  |  |  |  |  |  |  |
| I prefer not to answer | 157 | 8.9 (5.4-14.5) | 186 | 19.9 (14.8-26.3) | 6 | 16.7 (2.2-64.1) | 7 | 42.9 (14.2-77.3) |  |  |  |  |  |  |  |  |

Prevalence estimations with 95% CI that do not overlap between categories of the covariate indicating statistical significance are highlighted in **bold**.

^a^ TGE AMAB = transgender or gender expansive assigned male at birth; TGE AFAB = transgender or gender expansive assigned female at birth. ^b^ Spanish Healthy Alimentation Index (IASE), using a cut-off of ≤80 points for unhealthy diet. ^c^ Alcohol Use Disorders Identification Test (AUDIT-C), using a cut-off of ≥3 points for hazardous drinking. ^d^ Compulsive Internet Use Scale (CIUS), with a cut-off of ≥28 points for problematic internet use.

Alt text: Table displaying the prevalence and 95% confidence intervals of worse overall health in the four groups of study (cisgender boys, cisgender girls, TGE males and TGE females), according to the different covariates (health-related behaviours and interpersonal relations).

**Supplementary Table 2. Prevalence of mental discomfort (% and 95% CI) according to health-related behaviours and interpersonal relationships covariates, stratified by gender identity and sex assigned at birth.**

|  | **Cisgender boys** | | **Cisgender girls** | | **TGE AMAB^a^** | | **TGE AFAB^a^** | |  |  |  |  |  |  |  |  |
| --- | --- | --- | --- | --- | --- | --- | --- | --- | --- | --- | --- | --- | --- | --- | --- | --- |
|  | **N** | **% (95% CI)** | **N** | **% (95% CI)** | **N** | **% (95% CI)** | **N** | **% (95% CI)** |  |  |  |  |  |  |  |  |
| **General** | 4441 | 17.5 (16.5-18.7) | 4542 | 41.2 (37.9-42.6) | 67 | 34.3 (24.0-46.4) | 127 | 58.3 (49.5-66.5) |  |  |  |  |  |  |  |  |
| **Health behaviours** | | | | | | | | |  |  |  |  |  |  |  |  |
| **Quality of diet (IASE≤80)** ^b^ |  |  |  |  |  |  |  |  |  |  |  |  |  |  |  |  |
| Healthy | 248 | 13.3 (9.6-18.1) | 504 | **35.5 (31.5-39.8)** | 3 | 66.7 (14.8-95.8) | 20 | 45.0 (25.2-66.6) |  |  |  |  |  |  |  |  |
| Unhealthy | 4193 | 17.8 (16.7-19.0) | 4038 | **41.9 (40.4-43.4)** | 64 | 32.8 (22.3-45.4) | 107 | 60.7 (51.1-69.6) |  |  |  |  |  |  |  |  |
| **Physical activity (≥1h/day)** |  |  |  |  |  |  |  |  |  |  |  |  |  |  |  |  |
| Yes | 2564 | **13.7 (12.4-15.1)** | 1700 | **34.6 (32.4-36.9)** | 36 | 36.1 (22.0-53.1) | 44 | 45.5 (31.4-60.3) |  |  |  |  |  |  |  |  |
| No | 1877 | **22.7 (20.9-24.7)** | 2842 | **45.1 (43.3-46.9)** | 31 | 32.3 (18.1-50.6) | 83 | 65.1 (54.1-74.6) |  |  |  |  |  |  |  |  |
| **Daily tobacco consumption** |  |  |  |  |  |  |  |  |  |  |  |  |  |  |  |  |
| No | 4211 | 17.2 (16.1-18.4) | 4187 | 40.8 (39.4-42.3) | 64 | 32.8 (22.3-45.4) | 116 | 56.9 (47.7-65.7) |  |  |  |  |  |  |  |  |
| Yes | 230 | 23.5 (18.4-29.4) | 355 | 45.1 (40.0-50.3) | 3 | 66.7 (14.8-95.8) | 11 | 72.7 (41.1-91.1) |  |  |  |  |  |  |  |  |
| **Cannabis consumption (last 30 days)** |  |  |  |  |  |  |  |  |  |  |  |  |  |  |  |  |
| No | 4178 | 17.4 (16.3-18.6) | 4293 | **40.3 (38.8-41.8)** | 61 | 32.8 (22.1-45.7) | 117 | 57.3 (48.1-66.0) |  |  |  |  |  |  |  |  |
| Yes | 263 | 19.4 (15.1-24.6) | 249 | **56.2 (50.0-62.3)** | 6 | 50.0 (16.4-83.6) | 10 | 70.0 (37.3-90.1) |  |  |  |  |  |  |  |  |
| **Risk alcohol consumption (AUDIT-C≥3) ^c^** |  |  |  |  |  |  |  |  |  |  |  |  |  |  |  |  |
| No | 3302 | 17.6 (16.4-19.0) | 3269 | **39.7 (38.0-41.4)** | 52 | 30.8 (19.6-44.7) | 99 | 57.6 (47.6-67.0) |  |  |  |  |  |  |  |  |
| Yes | 1139 | 17.3 (15.2-19.6) | 1273 | **44.9 (42.2-47.7)** | 15 | 46.7 (23.7-71.1) | 28 | 60.7 (41.8-76.9) |  |  |  |  |  |  |  |  |
| **Compulsive screen and digital entertainment use (CIUS≥28) ^d^** |  |  |  |  |  |  |  |  |  |  |  |  |  |  |  |  |
| No | 3659 | **14.9 (13.8-16.1)** | 3258 | **35.5 (33.9-37.2)** | 50 | 34.0 (22.1-48.3) | 86 | 53.5 (42.8-63.8) |  |  |  |  |  |  |  |  |
| Yes | 782 | **29.9 (26.8-33.2)** | 1284 | **55.5 (52.7-58.2)** | 17 | 35.3 (16.5-60) | 41 | 68.3 (52.6-80.7) |  |  |  |  |  |  |  |  |
| **Interpersonal relations** | | | | | | | | |  |  |  |  |  |  |  |  |
| **Family relationships** |  |  |  |  |  |  |  |  |  |  |  |  |  |  |  |  |
| Good or very good | 3956 | **14.1 (13.1-15.2)** | 3507 | **32.6 (31.1-34.2)** | 50 | **24.0 (14.0-38.0)** | 76 | **47.4 (36.4-58.6)** |  |  |  |  |  |  |  |  |
| Regular/bad/very bad | 485 | **45.6 (41.2-50.0)** | 1035 | **70.0 (67.2-72.8)** | 17 | **64.7 (40.0-83.5)** | 51 | **74.5 (60.7-84.7)** |  |  |  |  |  |  |  |  |
| **Bullying victimization** |  |  |  |  |  |  |  |  |  |  |  |  |  |  |  |  |
| No | 3809 | **15.2 (14.1-16.4)** | 3686 | **37.5 (35.9-39.1)** | 47 | 36.2 (23.6-51.0) | 84 | 50.0 (39.4-60.6) |  |  |  |  |  |  |  |  |
| Yes | 632 | **31.6 (28.1-35.4)** | 856 | **57.0 (53.7-60.3)** | 20 | 30.0 (13.9-53.2) | 43 | 74.4 (59.3-85.3) |  |  |  |  |  |  |  |  |
| **Any sexual violence** |  |  |  |  |  |  |  |  |  |  |  |  |  |  |  |  |
| No | 3885 | **15.8 (14.7-17.0)** | 1976 | **32.7 (30.7-34.8)** | 45 | 28.9 (17.4-43.9) | 45 | 51.1 (36.7-65.4) |  |  |  |  |  |  |  |  |
| Yes | 399 | **30.3 (26.0-35.0)** | 2380 | **47.2 (45.2-49.2)** | 16 | 56.3 (32.0-77.9) | 75 | 64.0 (52.5-74.1) |  |  |  |  |  |  |  |  |
| Prefer not to answer | 157 | 28.0 (21.6-35.6) | 186 | 54.3 (47.1-61.3) | 6 | 16.7 (2.2-64.1) | 7 | 42.9 (14.2-77.3) |  |  |  |  |  |  |  |  |

Prevalence estimations with 95% CI that do not overlap between categories of the covariate indicating statistical significance are highlighted in **bold**.

^a^ TGE AMAB = transgender or gender expansive assigned male at birth; TGE AFAB = transgender or gender expansive assigned female at birth. ^b^ Spanish Healthy Alimentation Index (IASE), using a cut-off of ≤80 points for unhealthy diet. ^c^ Alcohol Use Disorders Identification Test (AUDIT-C), using a cut-off of ≥3 points for hazardous drinking. ^d^ Compulsive Internet Use Scale (CIUS), with a cut-off of ≥28 points for problematic internet use.

Alt text: Table displaying the prevalence and 95% confidence intervals of mental discomfort in the four groups of study (cisgender boys, cisgender girls, TGE AMAB and TGE AFAB), according to the different covariates (health-related behaviours and interpersonal relations).

**Supplementary Table 3. Analysis of the association of worse overall health with gender identity and sex assigned at birth.**

|  | **Model 1** ^a^ | | **Model 2 (behaviours)**^b^ | | **Model 3 (relations)** ^c^ | | **Model 4** ^d^ | |
| --- | --- | --- | --- | --- | --- | --- | --- | --- |
|  | **PR_adj_** | **95% CI** | **PR_adj_** | **95% CI** | **PR_adj_** | **95% CI** | **PR_adj_** | **95% CI** |
| **Gender identity** |  |  |  |  |  |  |  |  |
| Cisgender boys | 1.00 |  | 1.00 |  | 1.00 |  | 1.00 |  |
| Cisgender girls | **2.16** | **(1.87 - 2.49)** | **1.89** | **(1.63 - 2.19)** | **1.73** | **(1.48 - 2.02)** | **1.61** | **(1.37 - 1.89)** |
| TGE AMAB ^e^ | **2.01** | **(1.14 - 3.52)** | 1.74 | (0.93 - 3.25) | 1.53 | (0.85 - 2.75) | 1.41 | (0.74 - 2.71) |
| TGE AFAB ^e^ | **3.90** | **(2.97 - 5.12)** | **3.44** | **(2.60 - 4.55)** | **2.66** | **(2.04 - 3.48)** | **2.62** | **(2.00 - 3.42)** |
| **Age (years)** | **1.10** | **(1.06 - 1.14)** | **1.05** | **(1.01 - 1.09)** | **1.11** | **(1.08 - 1.16)** | **1.08** | **(1.04 - 1.12)** |
| **Socioeconomic position** |  |  |  |  |  |  |  |  |
| Low | 1.00 |  | 1.00 |  | 1.00 |  | 1.00 |  |
| Medium | **0.57** | **(0.50 - 0.66)** | **0.58** | **(0.51 - 0.67)** | **0.64** | **(0.56 - 0.73)** | **0.64** | **(0.56 - 0.73)** |
| High | **0.45** | **(0.39 - 0.53)** | **0.48** | **(0.41 - 0.56)** | **0.54** | **(0.46 - 0.63)** | **0.55** | **(0.47 - 0.65)** |
| **Migration status** |  |  |  |  |  |  |  |  |
| From Spain | 1.00 |  | 1.00 |  | 1.00 |  | 1.00 |  |
| Second-generation migrant | **1.36** | **(1.17 - 1.57)** | **1.33** | **(1.15 - 1.54)** | **1.26** | **(1.09 - 1.45)** | **1.25** | **(1.08 - 1.44)** |
| First-generation migrant | **1.31** | **(1.07 - 1.62)** | **1.32** | **(1.07 - 1.62)** | **1.29** | **(1.05 - 1.59)** | **1.29** | **(1.05 - 1.58)** |
| Missing | **1.31** | **(1.01 - 1.70)** | **1.30** | **(1.01 - 1.67)** | 1.14 | (0.89 - 1.47) | 1.16 | (0.91 - 1.48) |
| **Sexual orientation** |  |  |  |  |  |  |  |  |
| Heterosexual | 1.00 |  | 1.00 |  | 1.00 |  | 1.00 |  |
| Homosexual | **2.00** | **(1.49 - 2.69)** | **1.78** | **(1.34 - 2.37)** | **1.49** | **(1.12 - 1.98)** | **1.43** | **(1.08 - 1.90)** |
| Bisexual | **2.12** | **(1.83 - 2.46)** | **1.86** | **(1.60 - 2.15)** | **1.66** | **(1.43 - 1.93)** | **1.55** | **(1.34 - 1.80)** |
| Other | **1.60** | **(1.36 - 1.89)** | **1.53** | **(1.30 - 1.80)** | **1.34** | **(1.15 - 1.57)** | **1.31** | **(1.12 - 1.53)** |
| **Quality of diet** |  |  |  |  |  |  |  |  |
| Healthy |  |  | 1.00 |  |  |  | 1.00 |  |
| Unhealthy |  |  | **1.35** | **(1.07 - 1.70)** |  |  | **1.32** | **(1.05 - 1.65)** |
| **Physical activity (≥1h/day)** |  |  |  |  |  |  |  |  |
| Yes |  |  | 1.00 |  |  |  | 1.00 |  |
| No |  |  | **1.43** | **(1.25 - 1.64)** |  |  | **1.41** | **(1.23 - 1.61)** |
| **Daily tobacco consumption** |  |  |  |  |  |  |  |  |
| No |  |  | 1.00 |  |  |  | 1.00 |  |
| Yes |  |  | **1.70** | **(1.40 - 2.07)** |  |  | **1.58** | **(1.30 - 1.92)** |
| **Cannabis consumption (last 30 days)** |  |  |  |  |  |  |  |  |
| No |  |  | 1.00 |  |  |  | 1.00 |  |
| Yes |  |  | **1.47** | **(1.20 - 1.80)** |  |  | **1.30** | **(1.06 - 1.59)** |
| **Compulsive screen and digital entertainment use (CIUS≥28)** |  |  |  |  |  |  |  |  |
| No |  |  | 1.00 |  |  |  | 1.00 |  |
| Yes |  |  | **1.77** | **(1.57 - 2.00)** |  |  | **1.53** | **(1.36 - 1.72)** |
| **Family relationships** |  |  |  |  |  |  |  |  |
| Very good or good |  |  |  |  | 1.00 |  | 1.00 |  |
| Regular, bad or very bad |  |  |  |  | **2.37** | **(2.09 - 2.69)** | **2.11** | **(1.86 - 2.40)** |
| **Bullying victimization** |  |  |  |  |  |  |  |  |
| No |  |  |  |  | 1.00 |  | 1.00 |  |
| Yes |  |  |  |  | **1.69** | **(1.48 - 1.92)** | **1.62** | **(1.42 - 1.84)** |
| **Any sexual violence** |  |  |  |  |  |  |  |  |
| No |  |  |  |  | 1.00 |  | 1.00 |  |
| Yes |  |  |  |  | **1.23** | **(1.07 - 1.42)** | **1.17** | **(1.01 - 1.35)** |
| Prefer not to answer |  |  |  |  | 1.28 | (0.99 - 1.65) | 1.25 | (0.97 - 1.61) |

Prevalence ratios (PR) that are statistically significant are highlighted in **bold**.

^a^ Model 1 was adjusted by age, socioeconomic position, migration status, and sexual orientation. ^b^ Model 2 was adjusted by the variables in model 1 and quality of diet, physical activity, daily tobacco consumption, last month cannabis consumption, and compulsive screen and digital entertainment use. ^c^ Model 3 was adjusted by the variables in model 1 and family relationships, bullying victimization and any sexual violence. ^d^ Model 4 was adjusted by all the variables in the previous models.

^e^ TGE AMAB = transgender or gender expansive assigned male at birth; TGE AFAB = transgender or gender expansive assigned female at birth.

Alt text: Table displaying the adjusted prevalence ratios and 95% confidence intervals of worse overall health in cisgender girls, TGE AMAB, and TGE AFAB, compared to cisgender boys. Model 1 includes as adjustment variables only sociodemographic variables, model 2 adds behaviours to model 1, model 3 adds relations to model 1, and model 4 is the final model with all the previous variables

**Supplementary Table 4. Analysis of the association of mental discomfort with gender identity and sex assigned at birth.**

|  | **Model 1** ^a^ | | **Model 2 (behaviours)**^b^ | | **Model 3 (relations)** ^c^ | | **Model 4** ^d^ | |
| --- | --- | --- | --- | --- | --- | --- | --- | --- |
|  | **PR_adj_** | **95% CI** | **PR_adj_** | **95% CI** | **PR_adj_** | **95% CI** | **PR_adj_** | **95% CI** |
| **Gender identity** |  |  |  |  |  |  |  |  |
| Cisgender boys | 1.00 |  | 1.00 |  | 1.00 |  | 1.00 |  |
| Cisgender girls | **2.13** | **(1.98 - 2.29)** | **1.97** | **(1.82 - 2.12)** | **1.79** | **(1.66 - 1.94)** | **1.71** | **(1.58 - 1.85)** |
| TGE AMAB ^e^ | **1.63** | **(1.20 - 2.23)** | **1.57** | **(1.14 - 2.17)** | 1.34 | (0.98 - 1.85) | 1.34 | (0.97 - 1.85) |
| TGE AFAB ^e^ | **2.40** | **(2.04 - 2.82)** | **2.20** | **(1.87 - 2.59)** | **1.78** | **(1.51 - 2.10)** | **1.73** | **(1.47 - 2.04)** |
| **Age (years)** | **1.05** | **(1.03 - 1.07)** | **1.04** | **(1.02 - 1.06)** | **1.06** | **(1.04 - 1.08)** | **1.05** | **(1.03 - 1.07)** |
| **Socioeconomic position** |  |  |  |  |  |  |  |  |
| Low | 1.00 |  | 1.00 |  | 1.00 |  | 1.00 |  |
| Medium | **0.82** | **(0.77 - 0.88)** | **0.84** | **(0.79 - 0.90)** | **0.89** | **(0.84 - 0.95)** | **0.90** | **(0.85 - 0.97)** |
| High | **0.62** | **(0.58 - 0.67)** | **0.64** | **(0.59 - 0.69)** | **0.71** | **(0.65 - 0.76)** | **0.72** | **(0.66 - 0.78)** |
| **Migration status** |  |  |  |  |  |  |  |  |
| From Spain | 1.00 |  | 1.00 |  | 1.00 |  | 1.00 |  |
| Second-generation migrant | **1.24** | **(1.15 - 1.34)** | **1.20** | **(1.11 - 1.29)** | **1.18** | **(1.10 - 1.26)** | **1.15** | **(1.07 - 1.23)** |
| First-generation migrant | **1.27** | **(1.14 - 1.42)** | **1.22** | **(1.09 - 1.36)** | **1.25** | **(1.13 - 1.39)** | **1.21** | **(1.08 - 1.34)** |
| Missing | **1.44** | **(1.28 - 1.62)** | **1.40** | **(1.25 - 1.58)** | **1.31** | **(1.16 - 1.47)** | **1.29** | **(1.14 - 1.46)** |
| **Sexual orientation** |  |  |  |  |  |  |  |  |
| Heterosexual | 1.00 |  | 1.00 |  | 1.00 |  | 1.00 |  |
| Homosexual | **1.50** | **(1.26 - 1.79)** | **1.40** | **(1.18 - 1.65)** | **1.22** | **(1.04 - 1.44)** | **1.19** | **(1.02 - 1.39)** |
| Bisexual | **1.59** | **(1.48 - 1.72)** | **1.51** | **(1.40 - 1.63)** | **1.32** | **(1.22 - 1.43)** | **1.30** | **(1.20 - 1.40)** |
| Other | **1.43** | **(1.32 - 1.55)** | **1.39** | **(1.28 - 1.50)** | **1.27** | **(1.17 - 1.37)** | **1.24** | **(1.15 - 1.35)** |
| **Quality of diet** |  |  |  |  |  |  |  |  |
| Healthy |  |  | 1.00 |  |  |  | 1.00 |  |
| Unhealthy |  |  | **1.13** | **(1.02 - 1.26)** |  |  | 1.11 | (1.00 - 1.23) |
| **Physical activity (≥1h/day)** |  |  |  |  |  |  |  |  |
| Yes |  |  | 1.00 |  |  |  | 1.00 |  |
| No |  |  | **1.23** | **(1.15 - 1.31)** |  |  | **1.22** | **(1.14 - 1.29)** |
| **Cannabis consumption (last 30 days)** |  |  |  |  |  |  |  |  |
| No |  |  | 1.00 |  |  |  | 1.00 |  |
| Yes |  |  | **1.17** | **(1.05 - 1.30)** |  |  | 1.03 | (0.93 - 1.15) |
| **Compulsive screen and digital entertainment use (CIUS≥28)** |  |  |  |  |  |  |  |  |
| No |  |  | 1.00 |  |  |  | 1.00 |  |
| Yes |  |  | **1.53** | **(1.44 - 1.63)** |  |  | **1.35** | **(1.27 - 1.44)** |
| **Family relationships** |  |  |  |  |  |  |  |  |
| Very good or good |  |  |  |  | 1.00 |  | 1.00 |  |
| Regular, bad or very bad |  |  |  |  | **1.95** | **(1.83 - 2.07)** | **1.86** | **(1.75 - 1.97)** |
| **Bullying victimization** |  |  |  |  |  |  |  |  |
| No |  |  |  |  | 1.00 |  | 1.00 |  |
| Yes |  |  |  |  | **1.44** | **(1.35 - 1.53)** | **1.39** | **(1.30 - 1.48)** |
| **Any sexual violence** |  |  |  |  |  |  |  |  |
| No |  |  |  |  | 1.00 |  | 1.00 |  |
| Yes |  |  |  |  | **1.20** | **(1.12 - 1.29)** | **1.17** | **(1.09 - 1.26)** |
| Prefer not to answer |  |  |  |  | **1.26** | **(1.11 - 1.43)** | **1.26** | **(1.11 - 1.43)** |

Prevalence ratios (PR) that are statistically significant are highlighted in **bold**.

^a^ Model 1 was adjusted by age, socioeconomic position, migration status, and sexual orientation. ^b^ Model 2 was adjusted by the variables in model 1 and quality of diet, physical activity, last month cannabis consumption, and compulsive screen and digital entertainment use. ^c^ Model 3 was adjusted by the variables in model 1 and family relationships, bullying victimization and any sexual violence. ^d^ Model 4 was adjusted by all the variables in the previous models.

^e^ TGE AMAB = transgender or gender expansive assigned male at birth; TGE AFAB = transgender or gender expansive assigned female at birth.

Alt text: Table displaying the adjusted prevalence ratios and 95% confidence intervals of mental discomfort in cisgender girls, TGE AMAB, and TGE AFAB, compared to cisgender boys. Model 1 includes as adjustment variables only sociodemographic variables, model 2 adds behaviours to model 1, model 3 adds relations to model 1, and model 4 is the final model with all the previous variables
